# Editorial: Perspectives in antibiotic resistance and new antimicrobial drugs: 2025

**DOI:** 10.3389/fcimb.2026.1889175

**Published:** 2026-06-11

**Authors:** George F. Araj, Costas C. Papagiannitsis, Elisabetta Buommino, Nasib Singh, Emanuela Roscetto

**Affiliations:** 1 Clinical Microbiology Laboratory, Department of Pathology and Laboratory Medicine, and Center for Infectious Diseases Research, American University of Beirut, Beirut, Lebanon; 2 Faculty of Medicine, School of Health Sciences, University of Thessaly, Larissa, Greece; 3 Department of Pharmacy, School of Medicine and Surgery, University of Naples Federico II, Naples, Italy; 4 Department of Microbiology, Akal College of Basic Sciences, Eternal University, Baru Sahib, Himachal Pradesh, India; 5 Department of Molecular Medicine and Medical Biotechnology, University of Naples Federico II, Naples, Italy

**Keywords:** antibiotic resistance, antimicrobials, biofilm, gram-negative bacteria, mycobacteria, β-lactamases

## Introduction

Antimicrobial resistance (AMR) has emerged as one of the most significant global public health crisis associated with increased morbidity, mortality, and healthcare costs in both developing and developed economies (Ho et al., 2025). The widespread and unregulated use of antibiotics has resulted in the rapid emergence and dissemination of resistance across bacterial populations. This Research Topic was launched with the objective of providing a platform to present recent advances in interdisciplinary research on AMR in bacteria, yeasts and moulds with emphasis on elucidating the diverse mechanisms of AMR (β-lactamases, carbapenemases, biofilms), antibiotic delivery, novel antimicrobial agents, and nanomaterial-based antimicrobials. The slow pace of new antibiotic discovery, along with the rapid emergence of AMR, has significantly intensified the challenge of developing effective alternative therapies against bacteria, fungi, viruses, protozoa and helminths (Oliveira Júnior et al., 2025; Moyo et al., 2026). In this direction, studies are presented in which applications of antimicrobial peptides, polystyrene nanoparticles, nano-emulsions, nano-composites, biofilm inhibitors, agents used in traditional medicine, novel antibiotic-β-lactamase inhibitor combinations, and antibiotic-delivery systems have been explored (Luchi et al., 2026).

The fourteen studies presented in the topic shed light on the progress made towards tackling the menace of AMR and devising alternate promising strategies having superior efficacy, lower toxicity and better cost-effectiveness against infectious diseases.

Mies et al. reported the potent antimicrobial action of two CLEC3A-derived peptides i.e. HT-47 and WRK-30 against the clinical strains of *Klebsiella pneumoniae, Acinetobacter baumannii, Candida albicans, Candida auris* and *Cryptococcus neoformans*. An extensive membrane damage and cellular disruption was found to be the mechanism mode of CLEC3A-derived peptides against *C. auris* cells. Further, the peptides also exhibited dose-dependent inhibition of biofilm formation by fungi pathogens. Their findings highlight the potential of novel peptides as therapeutic candidates for combating antimicrobial resistance in bacteria and fungi.

The study by Zheng et al. deals with the worldwide problem of microplastics and nanoplastics and their impact on human health. Unexpectedly, the study reveals that polystyrene nanoparticles (PS-NPs) can reduce the virulence of *C. neoformans*. The research shows that PS-NP exposure improves survival in infected mice by inhibiting fungal capsule formation and decreasing the expression of ARG1 mRNA, which encodes a key factor that suppresses T-cell-mediated antifungal immunity. These changes are attributed to mitochondrial dysfunction within the fungus, including increased oxidative stress and metabolic disruptions. In brief, the results suggest that PS-NPs impair the pathogen’s ability to defend itself by damaging its mitochondrial integrity. These results pose an intriguing problem, regarding the long-term persistence and toxicity of PS-NPs in the environment. Their accumulation in natural environments could disrupt fungal community structures, potentially favouring certain species while inhibiting others and thus contributing to fungal resistance.

Yu et al. investigated the physicochemical properties, antifungal activity, mechanisms of action, and toxicity of chitosan-based nanocomposites containing silver nanoparticles (Chi-AgNPs) against emerging azole-resistant *Aspergillus fumigatus*. These nanocomposites proved to be superior to the individual components due to greater stability and uniformity of the nanoparticles, as well as the strong synergistic combination of chitosan’s membrane- targeting action and silver’s ability to generate ROS, which leads to severe damage to the fungal cell wall. Although further studies are needed, the results provide a solid basis for proposing chitosan–silver nanocomposites as a promising therapeutic alternative for combating resistant fungal infections.

Sharma et al. analysed the nature and role of bacterial biofilms in antimicrobial resistance and chronic infections, emphasising that they represent one of the major challenges facing modern medicine. The paper also discussed various biofilm-combating approaches, including quorum sensing inhibitors, biofilm-degrading agents and nanoparticles to enhance drug penetration, as well as innovative methods such as phage therapy and CRISPR-Cas-based gene editing tools. The most promising strategies appear to be those that combine multiple therapeutic approaches. However, many of these technologies are still in the experimental phase. Overall, the authors emphasised that a thorough understanding of biofilm biology, combined with innovative technologies, is necessary to address biofilm-related challenges in the medical field and to realise their potential in industrial and environmental applications.

Qiu et al. systematically analysed six mechanisms of bacterial resistance, noting that these mechanisms do not act in isolation but are regulated and coordinated by quorum sensing. The authors highlighted that quorum sensing represents a promising therapeutic target for reducing bacterial resistance and, in this context, emphasized the potential of traditional Chinese medicine (TCM) to counteract these mechanisms. The complex composition of TCM, which acts on multiple targets simultaneously, makes it more difficult for resistance to develop; the authors noted that its combined use with antibiotics not only reduces drug dosage but also minimizes side effects. The paper also explored the impact on antibiofilm efficacy of the various administration methods currently used in TCM and presented new methods under investigation, offering perspectives for future research in this field.

Cui et al. investigated the antibacterial susceptibility and molecular characteristics of vancomycin resistant *Enterococcus faecium* and *Enterococcus faecalis* from China. Multidrug-resistance against glycopeptides, β-lactams, fluoroquinolones and erythromycin was reported in 75% of isolates. Their findings revealed ST80-CT8524 as the most common cluster among the isolates along with co-existence of *vanA* and *vanM* genes.

In another study, Bai et al. reported the antibacterial and antibiofilm activity of isochlorogenic acid C, a novel plant-derived phenolic compound against *Escherichia coli*. On the basis of staining, microscopic and molecular analysis, isochlorogenic acid C was found to inhibit the biofilm formation of *E. coli* by decreasing the production of extracellular polysaccharides. In addition, downregulation of genes associated with c-di-GMP synthesis while upregulation of genes involved in c-di-GMP degradation was observed indicating inhibition of bacterial motility.

Kamau et al. conducted a multi-centre retrospective study in Hawaii, Washington, and Texas to investigate the prevalence of *bla*_OXA-1_ and *bla*_TEM-1_ in more than 400 isolates of *E. coli* and *Klebsiella pneumoniae* and to establish its correlation with susceptibility to piperacillin/tazobactam. This study highlighted the role of *bla*_OXA-1_ gene in conferring resistance to piperacillin/tazobactam in *E. coli* and *K. pneumoniae* isolates. The complexity of antibiotic resistance in these pathogens was underscored by the association between specific aminoglycoside resistance genes and gentamicin resistance. Overall, these findings underline the need for local epidemiological data and genetic profiling to guide effective antibiotic therapy.

Fan and Chen described an unusual case of multidrug-resistant *Myroides odoratimimus* infection in a cancer patient. The isolate exhibited extensive multidrug resistance mediated by *bla*_MUS-1_ gene. Interestingly, urine alkalinization improved the efficacy of minocycline. Their study offered novel insights into the management of *M. odoratimimus* infections in oncology settings.

Qian et al. investigated the clinical and microbiological efficacy of a novel drug combination of ceftazidime/avibactam (CAZ/AVI) against MDR *Pseudomonas aeruginosa* in a single-centre retrospective observational study. Study findings indicated superior clinical efficacy of CAZ/AVI, however its administration as combination therapy fails to impart benefits over monotherapy thus warranting need for further findings to substantiate the drug combination therapy.

In search of novel antimicrobials, Lei et al. reported the potent anti-mycobacterial activity of curcumin-cinnamon essential oil nanoemulsion against *Mycobacterium smegmatis* and *Mycobacterium tuberculosis.* The antimicrobial action was attributed to cell membrane damage resulting in protein leakage. At the same time, curcumin-cinnamon essential oil nanoemulsion had no detrimental effects on macrophage viability and thus holds a promise as a nature-derived and safer alternate for the treatment of the mycobacterial infections.

Maxwel A.A. provided opinion on local microenvironment, Ultrasound-responsive microbubbles in recalcitrant infections. These infections resulted from local microenvironment and pathogen phenotype-such as acidity, hypoxia, and biofilm tolerance rather than drug or dosing failures. Research indicated that the efflux inhibition and physical biofilm disruption by niche-directed technologies can resensitize pathogens to antimicrobial treatment. In this direction, ultrasound-responsive microbubbles uniquely combine real-time imaging with physical disruption and targeted drug delivery and offer promising solutions. Finally, localized delivery of potent molecules can be enabled by antibiotic salvage therapy using these transformative technologies.

Tao et al. investigated the genomic characteristics of carbapenem-resistant *Enterobacter* strains from burn patients for the co-occurrence of *bla*_NDM-1_ and *mcr-9* genes. Their findings revealed three *E. hormaechei* strains of sequence type 97 co-harbouring both the genes which are localized on the plasmids. These findings highlight potential risk of transmission of these resistance genes by mobile genetic elements and warrant effective implementation of genomic surveillance in hospital environment.

In a systematic review article, Zhang et al. provided a detailed analysis of resistance mechanisms, alternate therapeutic strategies, diagnostic tools and environmental reservoirs of carbapenem-resistant Gram-negative bacteria. Further, cefiderocol, sulbactam-durlobactam, and imipenem-relebactam were found to be promising antimicrobial agents. Their outcomes indicated the urgent need of multidisciplinary research and strengthening of global collaboration on cutting-edge research on AMR.

## Conclusion

In conclusion, the fourteen studies presented in this Research Topic emphasizes the significant developments towards effective, affordable and safer antimicrobial therapies against Gram-negative bacteria, Gram-positive bacteria, mycobacteria, yeasts and moulds for advancing our understanding of antimicrobial chemotherapy. The advancements in the field of novel antimicrobials and effective anti-infective therapy reflect the changing paradigm of renewed focus on medical interventions inspired from traditional medicine systems and safer nanotechnology-based antimicrobial delivery systems. Overall, this Research Topic highlights the collaborative and shared commitment of clinicians and researchers to a common goal of combatting and curtailing the threat of AMR.
